# Obstruction of the Intestinal Canal by a Gall Stone

**Published:** 1875-07-01

**Authors:** H. A. Johnson

**Affiliations:** No. 4 Sixteenth Street, Chicago


### MEDICAL EXAMINER.

A

*Semi-Monthly Journal of Medical Sciences.*

EDITED BY N. S. DAVIS, M.D., AND F. H. DAVIS, M.D.

No. XIII. CHICAGO, JULY 1, 1875. VOL. XVI.

### Original Communications.

### OBSTRUCTION OF THE INTESTINAL CANAL BY A GALL STONE.

REPORTED BY H. A. JOHNSON, M.D.

G. R. R., æt. 57, merchant, previously in good health, had, during the fall of 1874, an attack of jaundice, with some perihepatitis, from which, however, he apparently recovered. On the 30th of May, 1875, he consulted me at my office, for some difficulty of the bladder. The urine was scanty and loaded with uric acid. There was some tenderness over the region of the bladder; no abdominal tenderness, or discomfort elsewhere; tongue clean, and bowels regular. He stated to me then that his health, since his recovery from the jaundice, had been excellent; "never better," were his words. I ordered for him a diuretic mixture consisting mainly of potassa acet. I heard nothing from him again till June 6th, when I was called to visit him at his house. I then learned that the urinary trouble was soon relieved, and that he had been attending to his business till the day before, the 5th. He had been suffering all that day from pain in the bowels. During the night of the 5th and 6th there had been persistent vomiting, and the pain, which was spasmodic in character, and located in the umbilical region, became more intense. The tongue was pointed, coated in the centre, the edges clean; the temperature normal, skin natural, pulse 75 regular, urine scanty. Bowels had not been moved since the 4th. He was very restless, vomiting frequently, and the pain had become unbearable. Mustard poultices and warm fomentations had been used, but without any benefit. I ordered a pill containing one-fourth of a grain of morphine to be given and repeated every two hours till relieved. In a few hours I saw him again. He had found some, but not decided, relief from the pain. The vomiting continued. I then ordered the following:

℞—Hydr. Chlorid. mit. gr. iv.

Sodæ bicarb. ℨ ii.

M.—Ft. pulv. No. 2.

One to be given immediately, and the other in three hours. He took them both, and I think retained them, but in the evening the abdominal pain, which had never entirely ceased, became more severe. I carefully searched for hernia, and found none. I then told Mr. R. that I feared mechanical obstruction of the bowels. Injections of warm water and salt were ordered, but without any effect, and at 11 P. M. a hypodermic injection of one-fourth of a grain of morphine was given. He was soon relieved of the pain and fell asleep.

June 7th.—Has slept about four hours during the night; says he is better; "shall be all right as soon as the bowels move." The injection of water and salt was repeated, bringing away small quantities of indurated fæces. The pain returned, and the vomiting became, during the afternoon, stercoraceous. There was also obstinate hiccough; I ordered a chloroform mixture which controlled the hiccough. Dr. Byford saw him with me in the evening. We injected about two quarts—as much as the bowel would hold—of saline solution, retaining it as long as possible by mechanical aid. It came away unchanged in character. At 11 P. M. repeated the hypodermic injection of morphine, and remained with him during the night.

June 8th.—He slept some, not as much as the night before, and when awake vomited — sometimes mucus alone and sometimes a fluid of the color and consistence of pea soup, and having a decided fæcal odor. Pain in the bowels less; but little tenderness upon pressure; not much tympanitis. Temperature has so far not been above 99½° in the rectum; the skin has not been hot—is now cool; the pulse has gradually increased in frequency; was 106 at 11 P. M., last night, about 100 this morning, small and weak. At 12½ P. M. with the concurrence of Dr. Byford, injected slowly as much air as the bowel could be made to hold, using mechanical aid to prevent its escape. The course of the colon was marked by great inflation, and there was a conical tympanitic enlargement of the abdomen just below the umbilicus.

As the air was expelled, about an ounce of a fluid, in color, consistence and odor like that vomited, followed it. After an hour, ordered small injections of beef tea.

At 3 P. M. Dr. N. S. Davis saw him with Dr. Byford and myself. Morphine, by the hypodermic method, as necessary to procure rest, fomentations to the abdomen, which had previously been used, as well as the beef tea injections, were continued. The beef tea was mostly retained, but what came away was mixed with a pea-soup fluid like that vomited.

June 9th—A. M.—Has been kept quiet by morphine; one-fourth grain given at midnight; has not slept much; the vomiting has been frequent and offensive; is very restless when the effect of the anodyne begins to pass off; pulse irregular; surface cool; is not able to retain even a half-teaspoonful of anything on the stomach; urine very scanty; is very thirsty. I told him to drink all the water he wanted. He took a large goblet full of cold water and said, "That goes to the spot." Did not vomit it. Soon after he drank a tumbler full of milk, and kept that also. He subsequently took during the day both milk and beef tea by the stomach, but vomited, especially during the afternoon and evening. The urine became more copious, but the pulse continued to grow weaker and to become more irregular. Carbonate of ammonia and milk, with brandy and beef tea were given and continued during the night. Artificial warmth and stimulating applications were made to the surface, but all without any apparent effect upon the circulation. Morphine was used about every six hours in the one-fourth grain doses. He slept but little, and was very restless; intelligence perfect.

June 10th.—A. M.—Passed a restless night, but has not suffered much from positive pain. The abdomen is not largely distended, but the peristaltic movements are active and constant. The surface was covered with a cold perspiration; the features pinched; the pulse became, during the day, imperceptible; the eyes sunken; the breathing short and spasmodic. The retching and vomiting of small quantities of mucus and offensive matters continued; was extremely restless, requiring care to keep him on the bed. The morphine seemed to produce but little effect. These symptoms all continued into the night of the 10th, his strength gradually failing, and he died about 2 A. M., June 11th.

Autopsy 12 hours after death, in presence of Dr. W. H. Byford.—Abdomen not largely distended; adipose tissue abundant. Upon opening the abdominal cavity a portion of the small intestines were found to be very dark and congested, but there was no plastic lymph upon the peritoneal surface, and but little serous effusion. The colon was empty and normal in appearance, with the exception of a portion in right hypochondriac region where it was adherent to the liver. The ilio-cæcal valve was not obstructed. The ileum was collapsed and natural in color for about four feet above the valve, at which point a large gall stone was found firmly grasped by, and plugging up the canal. Above the obstruction the bowel was distended, containing a fluid identical in appearance with that which had been vomited. There was a marked difference in the color and consistence of the walls of the bowel above and below the obstruction. Just above at several points there was commencing gangrene. Upon tracing the small intestine up, the duodenum was found firmly attached, as was also the colon to the inferior surface of the right lobe of the liver. This whole gland was small in size, and no gall bladder could be found, but there existed in the substance of the liver, at the region of the gall bladder, a cavity containing about a half an ounce of pus not colored by bile. There was a large opening, sufficient to admit two fingers, from this cavity into the duodenum. The stomach contained about a pint of fluid. No other organs were examined.

The gall stone was firmly impacted in the bowel, is an inch and a quarter in length, with its extremities slightly rounded, and measures three and one quarter inches in circumference. It is very firm, and the surface was gritty to the touch.

REMARKS.

1. Would repeated inflation by distending the bowel below and perhaps around the gall stone have aided its descent? There seemed to have been some fluid from above the obstruction escaping around it at the time, or following the inflation that was practiced.

2. The general peritoneal inflammation was much less than usually exists in cases of obstruction by biliary calculus.

3. The presence of a pus-producing cavity in the location of the gall bladder, and the ulceration of the walls of the duodenum adherent to it, seems to have produced but little disturbance to the general health, if it existed before the 30th of May. The adhesion probably dated back to the attack of jaundice and perihepatitis in the fall of 1874.

4. Was the disturbance of the urinary secretion on the 30th of May connected in any way with the escape of the calculus from the cavity found in the location of the gall bladder into the duodenum?

5. Of five hundred cases of death from obstruction, collected by Dr. William Brinton, only 4.8 per cent. are from gall stones, and of these there were four times as many women as men. Their ages were over 50 years. The gall stones, with hardly an exception, became arrested in the jejunum or ileum.

6. Of these five hundred deaths, 43 per cent. were from intussusception, 17 per cent. were from stricture, 27.2 per cent. were from strangulation, 8 per cent. were from torsion, 4.8 per cent. were from gall stones.

7. Dr. John Syke Bristow, the author of the article on "Obstruction of the Bowels," in Reynolds' *System of Medicine*, limits the possible utility of an operation for relief to cases of strangulation and intussusception. In the other causes of obstruction, he says "an operation must, from the very nature of things, be at least useless." In reflecting upon this case, the query presents itself to my mind whether it would not have been possible, by the means of a strong needle inserted through the intestinal wall below the obstruction, to have so broken down the calculus that the fragments might have passed along the bowel.

As cases of intussusception, strangulation, and gall stones constitute 75 per cent. of all cases, and as it probably would sometimes happen that in operating for supposed intussusception or strangulation, that the cause of obstruction would be found to be a gall stone, I think the effort should be made to break it down, as suggested above, rather than the abandonment of the case when the operation had proceeded thus far.

No. 4 Sixteenth Street, Chicago, June 12th, 1875.

